# Lung cancer risk and exposure to air pollution: a multicenter North China case–control study involving 14604 subjects

**DOI:** 10.1186/s12890-023-02480-x

**Published:** 2023-05-24

**Authors:** Daojuan Li, Jin Shi, Di Liang, Meng Ren, Yutong He

**Affiliations:** Cancer Institute, the Fourth Hospital of Hebei Medical University, No.12 Jiankang Road, Changan district, Shijiazhuang, 050011 Hebei Province China

**Keywords:** Lung cancer, Case–control, Air pollution, Never-smokers, Nomogram model

## Abstract

**Background:**

For North Chinese lung cancer patients, there is limited study on the distribution of air pollution and smoking related features based on analyses of large-scale, high-quality population datasets. The aim of the study was to fully analyze risk factors for 14604 Subjects.

**Methods:**

Participants and controls were recruited in 11 cities of North China. Participants’ basic information (sex, age, marital status, occupation, height, and weight), blood type, smoking history, alcohol consumption, history of lung-related diseases and family history of cancer were collected. PM2.5 concentration data for each year in each city of the study area from 2005 to 2018 were extracted based on geocoding of each person's residential address at the time of diagnosis. Demographic variables and risk factors were compared between cases and matched controls using a univariate conditional logistic regression model. Multivariate conditional logistic regression models were applied to estimate the odds ratio (OR) and 95% confidence interval (CI) for risk factors in univariate analysis. The nomogram model and the calibration curve were developed to predict lung cancer probability for the probability of lung cancer.

**Results:**

There was a total of 14604 subjects, comprising 7124 lung cancer cases and 7480 healthy controls included in the study. Marital status of unmarried persons, people with a history of lung-related disease, corporate personnel and production /service personnel were protective factors for lung cancer. People younger than 50 years old, people who were smoking and quit smoking, people who had been drinking consistently, people with family history of cancer and PM2.5 exposure were proven to be a risk factor for lung cancer. The risk of lung cancer varied with sex, smoking status and air pollution. Consistent alcohol consumption, persistent smoking and smoking quit were risk factors for lung cancer in men. By smoking status, male was risk factor for lung cancer in never smokers. Consistent alcohol consumption added risk for lung cancer in never smokers. The combined effects of PM2.5 pollution exposure and ever smoking aggravated the incidence of lung cancer. According to air pollution, lung cancer risk factors are completely different in lightly and heavily polluted areas. In lightly polluted areas, a history of lung-related disease was a risk factor for lung cancer. In heavily polluted areas, male, consistent alcohol consumption, a family history of cancer, ever smokers and smoking quit were all risk factors for lung cancer. A nomogram was plotted and the results showed that PM2.5 was the main factor affecting the occurrence of lung cancer.

**Conclusions:**

The large-scale accurate analysis of multiple risk factors in different air quality environments and various populations, provide clear directions and guidance for lung cancer prevention and precise treatment.

## Background

According to the Globocan 2020, lung cancer was the second common cancer incidence and the leading cause of mortality, with 2.2 million new lung cancer cases and 1.8 million deaths in the world [1]. Lung cancer was also the leading cause of incidence and mortality in North China (Hebei province) [2, 3]. Many case–control and cohort studies have shown that the risk for developing lung cancer includes cigarette smoking, alcohol drinking, age, PM2.5 exposure, occupational exposures, sex, race, related lung disease and family history of cancer which are important contributors [4–8]. However, for North Chinese lung cancer patients, there is limited study on the distribution of air pollution and smoking related features based on analyses of large-scale, high-quality population datasets. The aim of the study was to fully analyze the key risk factors for 7124 North Chinese patients with lung cancer and 7480 healthy controls.

## Methods

### Study subjects

Participants were recruited between March 15th, 2005, and June 7th, 2018, in Shijiazhuang City, Baoding City, Tangshan City, Handan City, Xingtai City, Cangzhou City, Hengshui City, Langfang City, Qinhuangdao City, Chengde City and Zhangjiakou City in North China. Among the cities, Qinhuangdao City, Chengde City and Zhangjiakou City were defined as the lightly polluted cities and other cities were severely polluted cities [9].

Cases and matched controls were from a large case–control study of lung cancer by the Fourth Hospital of Hebei Medical University. Cases were newly-diagnosed and histologically confirmed primary lung cancer patients for the first time between March 15th, 2005, and June 7th, 2018, and had not undergone surgery, radiotherapy, or chemotherapy in the other hospital. Patients residing in local areas and with no other cancers.

Healthy controls without a history of cancer were recruited. Controls were frequency-matched on age (± 5 years), sex, and ethnicity with cases group. All participants provided written informed consent prior to participation in the study. This study was approved by the Ethics Committee of the Fourth Hospital of Hebei Medical University.

### Data collection and measurements

An expert group was established. Experts in various fields such as clinical medicine, epidemiology, health statistics, cancer registration, and specialized persons had long been engaged in clinical data collection. An expert seminar was held to demonstrate the study design and conduct technical guidance and quality control. The training meeting was held. Investigators were selected by the project site according to the workload, and the project team uniformly conducted technical training. All investigators strictly followed the standardized operating procedures. Experts from the project team visited the project sites at least once a year. The investigators conducted questionnaires on participants and controls. Participants’ basic information (sex, age, marital status, occupation), smoking history, alcohol consumption, history of lung-related diseases and family history of cancer were collected.

**Fig. 1:**
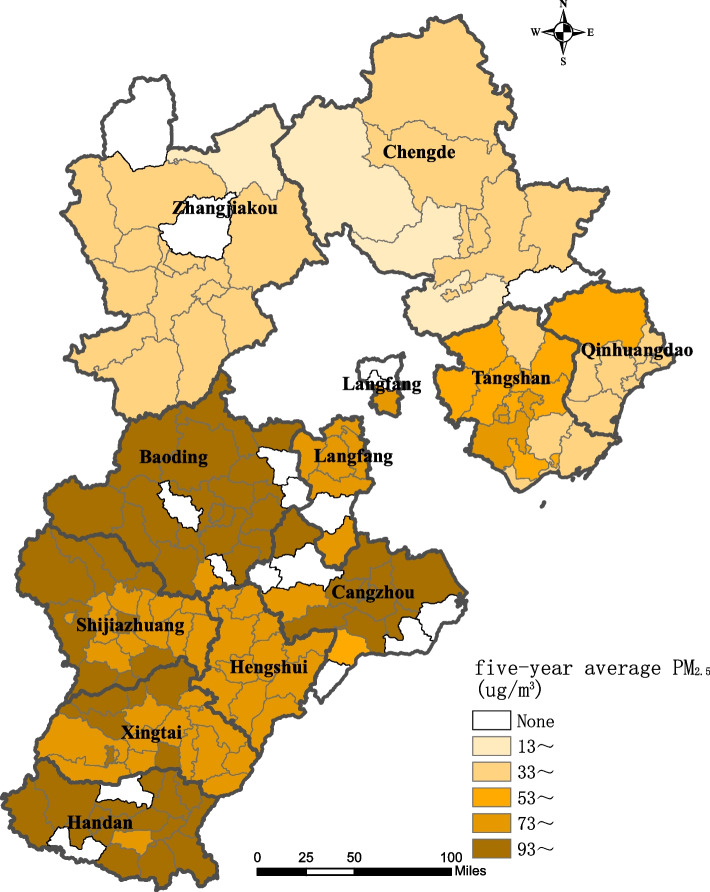
Five-year average concentration distribution of PM2.5 in 11 prefectures and cities

The PM < 2.5 microns in aerodynamic diameter (PM2.5) concentration data from 2005–2018 analyzed in this study were collected from the Atmospheric Composition Analysis Group of Dalhousie University [10], which has great accuracy and reliability as it had been corrected with global station-based observation values based on the geographically weighted regression model [11], with an R^2^ value of 0.817 and had been used in many studies [12–14]. The assessment of exposure to PM2.5 in this study was evaluated and the resulting annual mean PM2.5 concentrations were consistent with in-of-sample cross-validation observations (*R*^2 ^= 0.722). Information on air pollution measures (PM2.5) concentration data for each year in each city of the study area from 2005 to 2018 were extracted based on geocoding of each person's registered residential address. This study plotted the 5-year mean concentration distribution of PM2.5 including case–control subjects(Fig. 1). Referring to the Chinese government revised national ambient air quality standards (NAAQS) for particulate matter (PM) in 2012 (GB3095-2012), PM2.5 values are grouped with a cutoff value of 75 μg/m^3^ into lightly and heavily polluted areas [15, 16].

General characteristics were assessed to determine whether the distribution of participants differed significantly. The accuracy of the nomogram was assessed using the Concordance Index (C-index). Calibration curve was used to compare the association between actual outcomes and predicted probabilities.

### Statistical analysis

Demographic variables and risk factors were compared between cases and matched controls using a univariate conditional logistic regression model. Multivariate conditional logistic regression models were applied to estimate the odds ratio (OR) and 95% confidence interval (CI) for risk factors in univariate analysis. The nomogram was constructed to predict the lung cancer probability based on independent significant variables. All data analyses were performed using Statistical Package for the Social Sciences (SPSS) (version 20, SPSS Inc., Chicago, IL, USA) and R software version 3.3.4 (R Foundation for Statistical Computing, Vienna, Austria). All statistical tests were two-sided, and a threshold of *p* < 0.05 was statistically significant for all the statistical tests.

## Results

### Characteristics of the participants

There was a total of 14604 subjects, comprising 7124 lung cancer cases and 7480 healthy controls included in the study. Males accounted 56.48% (4103 cases for the case group, and 4145 cases for the control group) and females 43.52% (3021 cases for the case group, and 3335 cases for the control group) of total participants. In age group, the proportion of cases and control was higher in 60–70 years old than other groups.

Participants who were younger than 40 years old had a lower probability of developing lung cancer (OR, 0.397; 95% CI, 0.344–0.459). For sex, male had a higher probability of developing lung cancer (OR, 1.093; 95% CI, 1.024–1.167) than female. Unmarried cut their risk for lung cancer (OR, 0.377; 95% CI, 0.288–0.492) while married increased their risk for lung cancer (OR, 1.397; 95% CI, 1.172–1.664). Lung related diseases enabled the reduction of lung cancer risk (OR, 0.351;95% CI, 0.311–0.396). About smoking status, the result showed that participants who were smoking (OR, 1.843; 95% CI, 1.710–1.986) and smoking quit (OR, 2.584; 95% CI, 2.230–2.995) had a higher risk of lung cancer than never smoking participants. As for alcohol consumption, the findings showed that participants who had been drinking consistently had a 79.4% increased risk of lung cancer (OR, 1.794; 95% CI, 1.654–1.947) compared with never drinking. A family history of cancer multiplied their risk for lung cancer (OR, 2.824; 95% CI, 2.562–3.113) visibly. Corporate personnel and production/service workers were at lower risk of developing lung cancer than agriculture/fishing workers. We analyzed PM2.5 exposure as a continuous variable, and the results showed that five-year average PM2.5 exposure increased their risk for lung cancer (OR, 1.060; 95% CI, 1.057–1.062) (Table 1).

**Table 1:** General characteristics of lung cancer case group and control group

Factor	Cases N(%)	Control N(%)	*P*-value	OR	95%CI	
**Age**			0.000			
< 40	349(4.9)	879(11.8)	0.000	0.397	0.344	0.459
40–50	1696(23.8)	1569(21.0)	0.132	1.081	0.977	1.196
50–60	1786(25.1)	1739(23.2)	0.600	1.027	0.930	1.135
60–70	1909(26.8)	1909(25.5)	1.000	1.000	0.907	1.103
≥ 70	1384(19.4)	1384(18.5)		1.00	reference	
**Sex**			0.000			
Male	4103(57.6)	4145(55.4)		1.093	1.024	1.167
Female	3021(42.4)	3335(44.6)		1.00	reference	
**Marriage**			0.000			
Unmarried	115(1.7)	424(5.7)	0.000	0.377	0.288	0.492
Married	6785(95.2)	6745(90.2)	0.000	1.397	1.172	1.664
Widowed/Divorce	224(3.1)	311(4.1)		1	reference	
**Related disease**			0.000			
Yes	394(5.6)	1070(14.3)		0.351	0.311	0.396
No	6730(94.4)	6410(85.7)		1.00	reference	
**Smoking situation**			0.000			
Smoking	2312(32.5)	1641(21.9)	0.000	1.843	1.710	1.986
Smoking Quit	567(7.9)	287(3.8)	0.000	2.584	2.230	2.995
Never smoke	4245(59.6)	5552(74.2)		1.00	reference	
**Alcohol consumption**			0.000			
Yes	1823(25.6)	1203(16.1)		1.794	1.654	1.947
No	5301(74.4)	6277(83.9)		1.00	reference	
**Family history of cancer**			0.000			
Yes	1541(21.6)	666(8.9)		2.824	2.562	3.113
No	5583(78.4)	6814(91.1)		1.00	reference	
**Profession**			0.000			
Corporate personnel	717(10.1)	3943(52.7)	0.000	0.334	0.301	0.372
Production/Service	3431(48.2)	2056(27.5)	0.000	0.601	0.559	0.646
Agriculture/fishing	2976(41.8)	1481(19.8)		1.00	reference	
Five-year average PM2.5	7124(48.8%)	7480(51.2%)	0.000	1.060	1.057	1.062

### Multivariate analysis of risk factors

It showed the association between risk factors and lung cancer in Table 2. Marital status of unmarried (OR, 0.678; 95% CI, 0.481–0.956), males (OR, 0.496; 95% CI,0.453–0.543), people with a history of lung-related disease (OR, 0.394; 95% CI, 0.341–0.456), corporate personnel and production (OR, 0.421; 95% CI, 0.371–0.478)/service personnel (OR, 0.784; 95% CI, 0.719–0.854) were protective factors for lung cancer. People younger than 40 years old (OR, 1.767; 95% CI,1.450–2.153) and 40–50 years old (OR, 1.630; 95% CI,1.436–1.850), people who were smoking (OR, 2.630; 95% CI, 2.364–2.925) and quit smoking (OR, 4.232; 95% CI, 3.505–5.110), people who had been drinking consistently (OR, 1.295; 95% CI,1.156–1.451), people with family history of cancer (OR, 2.785; 95% CI,2.462–3.150) and PM2.5 pollution exposure (OR, 1.062; 95% CI,1.059–1.065) were proven to be a risk factor for lung cancer.

**Table 2:** Estimated risks of lung cancer associated with multivariate factors

**Factor**	***P*-value**	**OR**	**95%CI**	
**Age**	0.000			
< 40	0.000	1.767	1.450	2.153
40–50	0.000	1.630	1.436	1.850
50–60	0.000	1.287	1.140	1.453
60–70	0.000	1.268	1.128	1.426
≥ 70		1.00	reference	
**Sex**	0.000			
Male		0.496	0.453	0.543
Female		1.00	reference	
**Marriage**	0.000			
Unmarried	0.027	0.678	0.481	0.956
Married	0.001	1.463	1.178	1.816
Widowed/Divorce		1.00	reference	
**Related disease**				
Yes	0.000	0.394	0.341	0.456
No		1.00	reference	
**Smoking situation**	0.000			
Smoking	0.000	2.630	2.364	2.925
Smoking Quit	0.000	4.232	3.505	5.110
Never smoke		1.00	reference	
**Alcohol consumption**	0.000			
Yes	0.000	1.295	1.156	1.451
No		1.00	reference	
**Family history of cancer**	0.000			
Yes	0.000	2.785	2.462	3.150
No		1.00	reference	
**Profession**	0.000			
Corporate personnel	0.000	0.421	0.371	0.478
Production/Service	0.000	0.784	0.719	0.854
Agriculture/fishing		1.00	reference	
**Five-year average PM2.5**	0.000	1.062	1.059	1.065

**Fig. 2:**
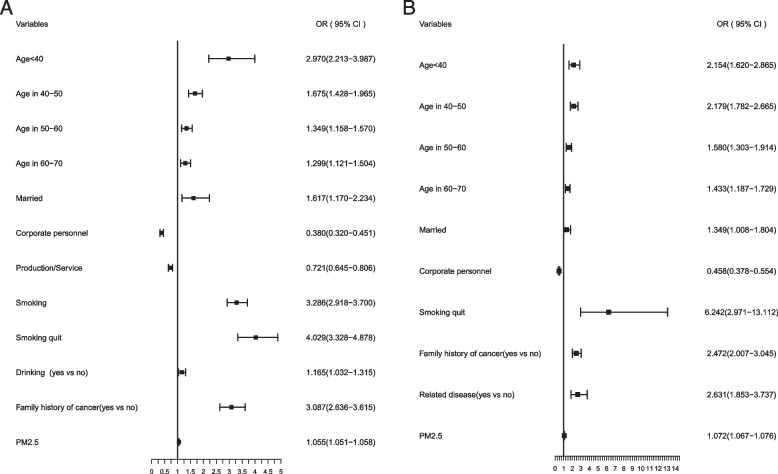
Multivariate risks of lung cancer by sex. **A** Multivariate risk factors model in males. **B** Multivariate risk factors model in females

We analyzed the impact of different variables on lung cancer stratifying by sex, smoking status, and air pollution. By sex, notable results were that there were big differences in smoking status for male and female. It may be due to the low smoking rate among Chinese women, persistent smoking (OR, 3.286; 95% CI, 2.918–3.700) and smoking quit (OR, 4.029; 95% CI, 3.328–4.878) were risk factors for lung cancer in men, while only smoking quit after smoking was a risk factor for lung cancer in women (OR, 6.242; 95% CI, 2.971–13.112), and the impact on women was greater than that in men. Consistent alcohol consumption added risk for lung cancer in men, but not in women. Men and women with a family history of cancer had 3.087-fold and 2.472-fold higher risk of developing lung cancer, respectively. PM2.5 pollution exposure was a risk factor for lung cancer in both men (OR, 1.055; 95% CI, 1.051–1.058) and women (OR, 1.072; 95% CI, 1.067–1.076). In addition, a history of lung-related disease was a risk factor for lung cancer in female (OR, 2.631; 95% CI, 1.853–3.737) (Fig. 2).

**Fig. 3:**
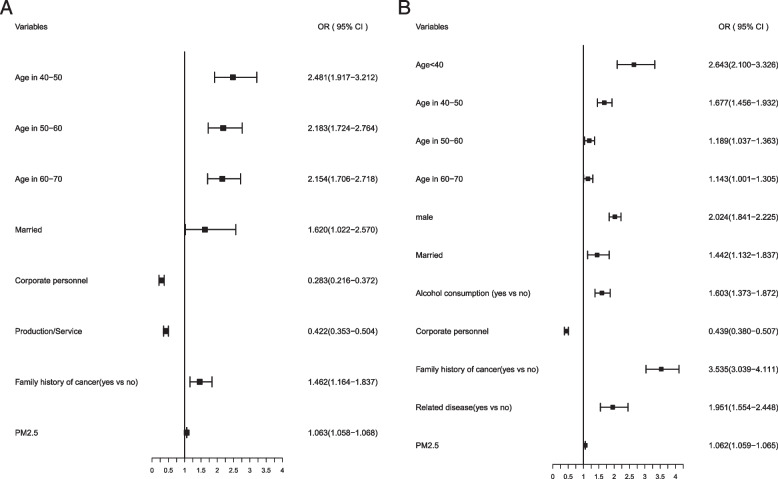
Multivariate risks of lung cancer by smoking. **A** Multivariate risk factors model in ever smokers. **B** Multivariate risk factors model in never smokers

By smoking status, men were more likely to develop lung cancer than women in never smokers. Consistent alcohol consumption added risk for lung cancer in never smokers. Never smokers with a family history of cancer had 3.535-fold higher risk of developing lung cancer than ever smokers with 1.462-fold. Among never smokers, a history of lung-related disease was a risk factor for lung cancer. Obviously, the combined effects of PM2.5 pollution exposure and ever smoking aggravated the incidence of lung cancer (OR, 1.063; 95% CI, 1.058–1.0.68) (Fig. 3).

**Fig. 4:**
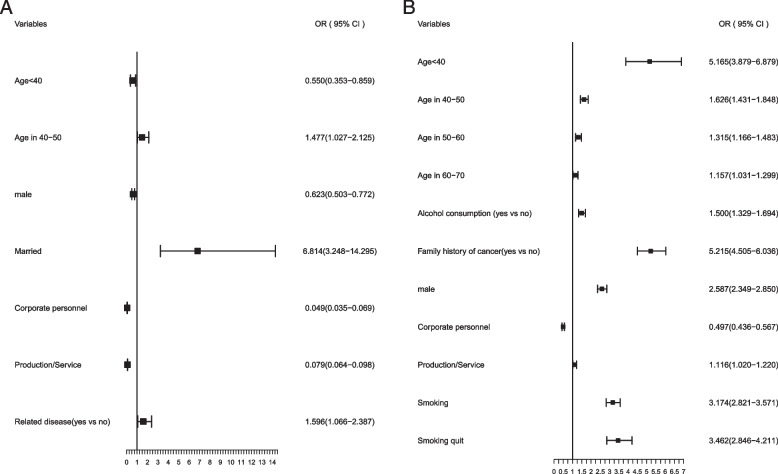
Multivariate risks of lung cancer by air pollution. **A** Multivariate risk factors model in lightly polluted areas. **B** Multivariate risk factors model in heavily polluted areas

According to air pollution, lung cancer risk factors are completely different in lightly and heavily polluted areas. In lightly polluted areas, a history of lung-related disease was a risk factor for lung cancer. In heavily polluted areas, male, consistent alcohol consumption, a family history of cancer, ever smokers and smoking quit were all risk factors for lung cancer (Fig. 4).

### Lung cancer nomogram

**Fig. 5:**
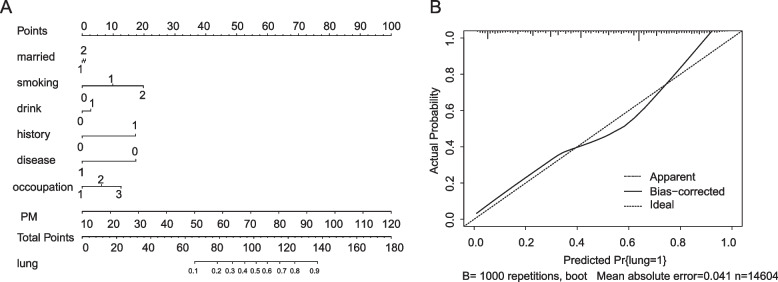
**A** A nomogram was used to predict the lung cancer probability **B** Validation of the nomogram—calibration plots for predicting lung cancer. In the marriage variable, 1 referred to unmarried and 2 referred to married. In smoking status, 0 represented never smoking, 1 represented being smoking, and 2 represented quitting smoking after smoking. In the drinking status, 0 represented not drinking, and 1 represented being drinking. In the family history of cancer, 0 represented having no family history of cancer, and 1 represented having a family history of cancer. In the lung related disease, 0 represented having no lung related disease, and 1 represented having lung related disease. In the occupation, 1 represented Corporate personnel, 2 represented Production/Service and 3 represented Agriculture/fishing

A nomogram was developed based on the significant factors identified in the log-rank model. In the nomogram estimation system, a weighted point value was attributed to each factor that implied a contribution to the lung cancer. We found that participants with higher scores had a higher chance of developing lung cancer than that observed in those with lower scores. The result showed that PM2.5 was the largest contributor to the occurrence of lung cancer, with the increase of PM2.5, the greater the possibility of lung cancer occurrence. The final nomogram model was developed to predict lung cancer probability and the calibration curve for the probability of lung cancer in the model was good. The C-statistic of the nomogram to predict lung cancer was 0.811 (95%CI, 0.805- 0.818) (Fig. 5).

## Discussion

Lung cancer has a high disease burden worldwide, second only to breast cancer. Lung cancer burden in North China was also heavy [2, 3]. A large multicenter case–control study of lung cancer risk factor in North China has not yet been reported. The study was to analyze cigarette smoking, alcohol drinking, age, PM2.5 exposure, profession, sex, marriage, related lung disease and family history of cancer for 7124 patients with lung cancer.

Our study found that people younger than 50 years old was a risk factor, indicating that maybe the age at onset of lung cancer tends to be younger. This was consistent with the findings of Ye T and Shi J et al. [17, 18]. Advancing the age for lung cancer screening may be necessary. Comparison of the odds of developing lung cancer between men and women was uncertain. This is different from the studies [19, 20]. Marital status of unmarried was protective factors for lung cancer while married was not. The unmarried people were relatively young, with 72.5% of unmarried people being under the age of 50 years old. The unmarried people lived a comfortable life with less the occupational stress and the social and economic pressure of supporting their families [21], and they were mentally happy. They had no the life pressure of middle-aged people who need to support the elderly. The phenomenon of young people not married seemed like more and more. According to data from the Seventh National Census [22], the total fertility rate in China reached a low of 1.3 in 2020, partly because more and more people don't want to get married. One study about demographic characteristics with lung cancer patients showed that the married proportions in Non-Hispanic white and Asian/Pacific Islander were highest with 54.5% and 66.1%, respectively. The overall Asian/Pacific Islander group was significantly more often married. Chinese lung cancer patients were more often married (70.4%), compared to Japanese, Hawaiian/Pacific Islander, Korean, and Vietnamese patients [23], which was consistent with our study. Lung related diseases enabled the reduction of lung cancer risk. One prospective study showed that a lung cancer screening criterion reflecting the severity of COPD may increase the sensitivity of the lung cancer screening program and reduce over-diagnosis of lung cancer [24]. It can prevent lung cancer through early screening, thereby reducing the incidence of lung cancer. Participants who were smoking and never smoking had a lower risk of lung cancer than participants who quit smoking. Cigarette smoking was a major risk factor for lung cancer, the hazard ratios for lung cancer were significantly higher in ever smokers than in never smokers [25, 26]. This is the same as our study. The difference in our results may be since the cut-off point and duration of smoking cessation were not accurately regulated when the participant information was collected. Most of the participants may have just quit smoking or had not quit smoking for a period that was conducive to good health. But the significant impact of smoking on the occurrence of lung cancer was undeniable. Consistent alcohol consumption increased risk for lung cancer. Some studied were like this study of the result [27, 28]. Our study suggested that a family history of cancer multiplied their risk for lung cancer. This is the same as the research result of Yu et al. [29]. We also found that corporate personnel and production /service personnel were protective factors for lung cancer.

As our results showed that air pollution and smoking status are key influencing factors for lung cancer, we performed a stratified analysis. For sex, the study showed that male smokers and drinkers were a high-risk group, and it’s very necessary to attach great importance to their lung cancer screening, and advocate early smoking and drinking cessation, healthy lifestyle and exercise. In female, a history of lung-related disease was a risk factor for lung cancer, so high attention was paid to the potential cancer risk brought by lung-related diseases to women, and further research was needed. For smoking status, male had a higher risk factor for lung cancer than female in never smokers. And consistent alcohol consumption added risk for lung cancer in never smokers. There was no clear evidence here that the combined effects of drinking and smoking increased lung cancer risk. Never smokers with a family history of cancer had 3.535-fold higher risk of developing lung cancer than ever smokers with 1.462-fold. In this study, women accounted for 57% of the non-smoking group and 15% of the smoking group. One research had shown that the association between family history and the lung cancer risk was identified among never-smokers (aOR 2.78, 95% CI 1.57 -4.90), but not among ever smokers (aOR 0.67, 95% CI 0.22–2.04). Unexpectedly, this study did not observe any significant associations between family history of lung/any cancer and lung cancer among ever-smokers in our study [30]. This suggested that although it was also known as a significant interaction between smoking status and family history of lung cancer, familial clustering of cancer was possibly not due to shared familial smoking habits among women.

For air pollution, age younger than 40 years old and male was the protective factor for lung cancer in lightly polluted areas. In lightly polluted areas, a history of lung-related disease was a risk factor for lung cancer. In areas with good air quality, people were more sensitive to lung-related diseases, and lung screening for this population needed to be strengthened. In lightly polluted areas, marriage was a higher risk factor for lung cancer. Tobacco remains the leading risk factor for lung cancer. PM2.5 exposure is less significant than smoking, which is possible, unless the study area is a very heavily polluted area. Obviously, this was the analysis result under the stratification of lightly polluted areas. In lightly polluted areas, 64% of people in cases and control groups did not smoke, which may be the reason for the smaller impact of smoking, and 85% of people were married. The unmarried people lived a comfortable life with less the occupational stress and the social and economic pressure of supporting their families. They had no the life pressure of middle-aged people who need to support the elderly. The phenomenon of young people not married seemed like more and more. The total fertility rate in China reached a low of 1.3 in 2020, partly because more and more people didn't want to get married. The final result analysis showed that marriage was a higher risk factor for lung cancer. Being younger than 40 years old, having a family history of cancer, and smoking or having smoked in the past were all higher risk factors for lung cancer in heavily polluted areas. It could be interpreted as: in heavily polluted areas, the age of onset of lung cancer was advanced, which maybe induce genomic instability in patients with a family history of cancer but it needed to be verified. Heavy pollution and smoking together did aggravate the risk of lung cancer. In heavily polluted areas, consistent alcohol consumption, a family history of cancer, ever smokers and smoking quit were all risk factors for lung cancer, which may imply the comprehensive effects of multiple factors. One study in the UK showed that the additive interaction between air pollution and a family history of cancer increased the risk of lung cancer [31]. The study in China found that the interaction effects of smoking and air pollutants increases this risk of lung cancer [32]. In the Nurses' Health Study cohort with two fully adjusted best-fit models that included smoking pack-years, smoking duration, time since cessation, and an age*pack-years interaction and average packs, smoking duration, and time since cessation, the results indicated that the HR per 10-μg/m3 increase in PM2.5 was 1.06 (95% CI: 0.90, 1.25) and 1.02 (95% CI: 0.87, 1.20)in the overall cohort and 1.35 (95% CI = 1.00, 1.82) and 1.27 (95% CI = 0.95, 1.71) among former smokers, respectively [33]. All these studies were highly consistent with this study. In addition, the nomogram and calibration curve were analyzed and plotted in the study and the results showed that PM2.5 was the main factor affecting the occurrence of lung cancer. Controlling the harm of air pollution to the human body, especially preventing the occurrence of lung cancer, was the most critical action.

The study accurately located the PM2.5 index of 14604 subjects, which was the first large-scale study on relation between PM2.5 pollution and lung cancer in North China. Indisputably, there were some deficiencies in our research. We should strictly control the duration time of smoking cessation. There may be stratified differences in the influence of the history of lung-related diseases, but there is no doubt that the history of lung-related diseases needs to be taken seriously, and physical examination items should be added. There were more risk factors, such as body mass index, diabetes, asbestos, diet, and exercise, which the study did not obtain. This study was a retrospective large scale population survey. For the body mass index, diet and exercise, it maybe not easy to collect and quantify. The number of people with diabetes and asbestos in the datasets is too small to analyze. In addition, the other co-pollutants other than the most important PM2.5 exposure was not access. It was indeed a deficiency of our research. But the acquisition of the PM2.5 exposure corresponding to 14604 subjects had been a significant and difficult breakthrough.

This study involves a large-scale survey of 14604 subjects in North China, and accurate analysis of multiple risk factors in different air quality environments and various populations, providing clear directions and guidance for lung cancer prevention and precise treatment.

## Data Availability

All data generated or analyzed during this study are included in this published article.
